# Aqua­bis(1*H*-benzimidazole-2-carboxyl­ato-κ^2^ *O*,*N* ^3^)zinc(II)

**DOI:** 10.1107/S1600536810015631

**Published:** 2010-05-08

**Authors:** Li Li Di, Yue Wang, Guo Wu Lin, Tao Lu

**Affiliations:** a Basic College Science, Laboratory of Pharmaceutical Chemistry, China Pharmaceutical University, Nanjing, People’s Republic of China

## Abstract

In the title compound, [Zn(C_8_H_5_N_2_O_2_)_2_(H_2_O)], the Zn^II^ ion is coordinated in each case by a carboxyl­ate O atom and an imidazole N atom from two different benzimidazole-2-carboxyl­ate (BIC) ligands and one water O atom in a trigonal-bipyramidal geometry. In the complex mol­ecule, the two benzimidazole planes are twisted, making a dihedral angle of 55.93 (11)°. The three-dimensional framework is organized by inter­molecular N—H⋯O hydrogen bonding and O—H⋯O inter­actions and π–π inter­actions between adjacent benzimidazole rings [centroid–centroid distance = 3.586 (3) Å].

## Related literature

For the biological activity of zinc complexes, see: Yoshikawa *et al.* (2001 ▶); Adachi *et al.* (2004 ▶). For the biological activity of benzimidazole derivatives, see: Shingalapur *et al.* (2009 ▶). For zinc *N*-heterocyclic or their carboxyl­ate complexes, see: He (2006 ▶); Li *et al.* (2007 ▶); Gao *et al.* (2005 ▶). For the structural index τ, see: Addison *et al.* (1984 ▶). For related structures, see: Liu *et al.* (2004 ▶); Lin (2006 ▶); Zhong *et al.* (2006 ▶).
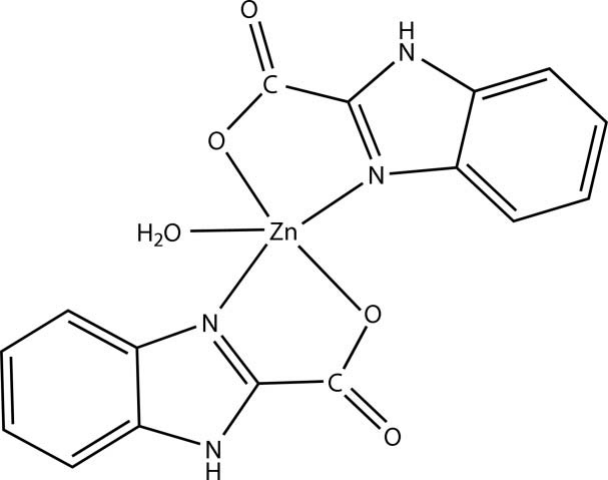


## Experimental

### 

#### Crystal data

[Zn(C_8_H_5_N_2_O_2_)_2_(H_2_O)]
*M* *_r_* = 405.67
Monoclinic, 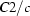

*a* = 25.9702 (15) Å
*b* = 10.0870 (6) Å
*c* = 16.7885 (10) Å
β = 129.21 (3)°
*V* = 3407.8 (14) Å^3^
*Z* = 8
Mo *K*α radiation
μ = 1.48 mm^−1^
*T* = 293 K
0.20 × 0.10 × 0.10 mm

#### Data collection

Enraf–Nonius CAD-4 diffractometer
Absorption correction: ψ scan (*ABSCOR*; Higashi, 1995 ▶) *T*_min_ = 0.757, *T*_max_ = 0.866
6178 measured reflections
3097 independent reflections
2368 reflections with *I* > 2σ(*I*)
*R*_int_ = 0.039
3 standard reflections every 120 min  intensity decay: 1%

#### Refinement

*R*[*F* ^2^ > 2σ(*F* ^2^)] = 0.039
*wR*(*F* ^2^) = 0.101
*S* = 0.99
3097 reflections
235 parameters
H-atom parameters constrained
Δρ_max_ = 0.56 e Å^−3^
Δρ_min_ = −0.56 e Å^−3^

### 

Data collection: *CAD-4 EXPRESS* (Enraf–Nonius, 1994 ▶); cell refinement: *CAD-4 EXPRESS*; data reduction: *XCAD4* (Harms & Wocadlo, 1995 ▶); program(s) used to solve structure: *SHELXS97* (Sheldrick, 2008 ▶); program(s) used to refine structure: *SHELXL97* (Sheldrick, 2008 ▶); molecular graphics: *SHELXTL* (Sheldrick, 2008 ▶); software used to prepare material for publication: *CrystalStructure* (Rigaku, 2002 ▶).

## Supplementary Material

Crystal structure: contains datablocks global, I. DOI: 10.1107/S1600536810015631/kp2256sup1.cif

Structure factors: contains datablocks I. DOI: 10.1107/S1600536810015631/kp2256Isup2.hkl

Additional supplementary materials:  crystallographic information; 3D view; checkCIF report

## Figures and Tables

**Table:** 

Zn1—O5	1.947 (3)
Zn1—N1	2.007 (3)
Zn1—N3	2.014 (2)
Zn1—O4	2.184 (2)
Zn1—O2	2.193 (2)

**Table:** 

O5—Zn1—N1	116.39 (12)
O5—Zn1—N3	115.42 (12)
N1—Zn1—N3	128.19 (11)
O5—Zn1—O4	93.18 (11)
N1—Zn1—O4	97.49 (10)
N3—Zn1—O4	79.46 (9)
O5—Zn1—O2	90.98 (11)
N1—Zn1—O2	79.56 (10)
N3—Zn1—O2	99.82 (10)
O4—Zn1—O2	175.67 (9)

**Table 2:** Hydrogen-bond geometry (Å, °)

*D*—H⋯*A*	*D*—H	H⋯*A*	*D*⋯*A*	*D*—H⋯*A*
N2—H2*A*⋯O1^i^	0.86	1.93	2.765 (4)	162
N4—H4*A*⋯O3^ii^	0.86	1.95	2.778 (4)	161
O5—H5*A*⋯O2^iii^	0.85	2.06	2.670 (4)	128
O5—H5*B*⋯O4^iv^	0.85	2.18	2.695 (5)	119

Symmetry codes: (i) 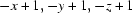; (ii) 
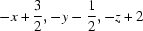
; (iii) 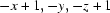; (iv) 
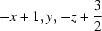
.

**Fig. 1.**
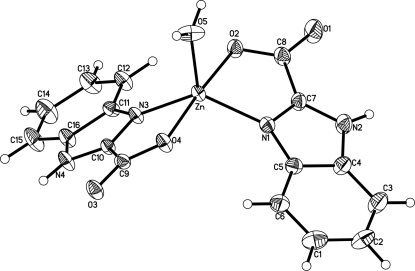
ORTEP drawing of (I) with the atomic numbering scheme. Ellipsoids for non-H atoms corresponding to 50 % probability.

**Fig. 2.**
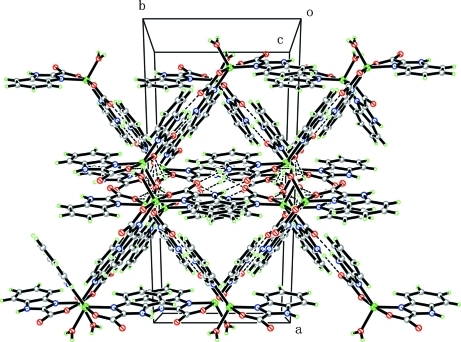
Packing drawing of (I) with the hydrogen bonds indicated by dashed lines.

## supplementary crystallographic information

### Comment

Zinc complexes with ligands such as amino acids, picolinic acid, vitamins, and allixin (isolated from garlic) are found to have high in vitro insulinomimetric activity and in vivo anti-diabetic activity (Yoshikawa *et al.*, 2001; Adachi *et al.*, 2004). Although many zinc N-heterocyclic or their carboxylate complexes have been reported(He, 2006; Li *et al.*, 2007; Gao *et al.*, 2005), those related to the benzimidazole-2-carboxylic acid(BICA) are reported rearly. Benzimidazole analogues are known to exhibit a wide variety of pharmacological properties; benzimidazole is an important pharmacophore and privileged structure in medicinal chemistry encompassing a diverse range of biological activities (Shingalapur *et al.*, 2009). In the viewpoint of constructing new functional complex, we prepared aqua(1*H*-benzo[d]imidazole-2-carboxylato-κ^2^*O*:*N*)-zinc(II) with the formula [Zn(C_8_H_5_N_2_O_2_)_2_(H_2_O)] (I) and report its structure.

In (I) Zn ^II^ coordination sphere is completed by carboxylato O atom and imidazole N atom from two different ligand molecules and one O atom from water (Fig. 1). The two BICA molecules are deprotonated to make the molecule neutral, then the BIC^-^ ligand behaves as a chelating unit that binds through the imidazole N atom and  carboxylato O atom, giving a five-membered chelate ring. The structural index τ (Addison *et al.* 1984) which represents the relative amount of trigonality of the five-coordination geometry as τ = 0 for a square pyramid and τ = 1 for a trigonal bipyramida in (I) is 0.791, therefore, the coordination geometry around Zn^II^ seems to be classified as a trigonal bipyramid rather than a square pyramid;  N3, O5, and N1 atoms form the equatorial trigonal plane indicated by the angle of O5-Zn-N1 and O5-Zn1-N3 being 116.39 (12) ° and 115.42 (12) °, respectively. The axial position occupy  O2 and O4 atoms; O4-Zn-O2 is the only combination with bonding angle close to 180 degrees. Therefore, the five-coordination geometry around Zn is classified as a distorted trigonal bipyramid with ZnN_2_O_3_ core. Compared with the other analogous zinc complexes, the bond distance of Zn^II^ and carboxylato O in (I) is in the normal range from 2.1445 (12) Å to 2.201 (3) Å (Table 1), while Zn—O5(water O atom) distance is significantly shorter than in the similar aqua zinc structures, such as 2.1565 (14) Å in [Zn(H_2_IDC)_2_(H_2_O)_2_] (where H_2_IDC is the 5-carboxy-1*H*-imidazole-4-carboxylate ligand (Liu *et al.*, 2004), 2.020 (3) Å in {[Zn(HIDC)(H_2_O)].0.5H_2_O}_n_ (HIDC is the imidazole-4,5-dicarboxylate ligand) (Lin, 2006) and 2.1491 (12) Å in [Zn(C_5_H_3_N_2_O_4_)_2_(H_2_O)_2_].C_10_H_8_N_2_(Zhong *et al.*, 2006).

The benzimidazole moiety in each ligand is nearly coplanar with the mean deviation from plane by 0.0059 Å and 0.0042 Å for ring C1—C7/N1/N2 and C10—C16/N3/N4, respectively. Around Zn^II^ two five-chelate rings are formed with slightly different conformations. The ring Zn1/N1/C7/C8/O2, adopts an envelope conformation with the deviation of Zn atom from the mean plane by 0.0686 (16) Å whereas the related ring Zn1/N3/C10/C9/O4 seems to be planar with the corresponding distance 0.0056 (14) Å. The dihedral angle of two benzimidazole groups around Zn^II^  is about 55.93 (11) ° leading to the intersection mode in the stack (Fig.2). In the ab plane, the whole zig-zag motif is bulit up, together with the π–π interactions between the adjacent benzimidazole planes along the axis a. The pairs of the two benzimidazole rings are oriented almost parallel and overlap face to face with the Cg···Cg distances of 3.586 (3) Å (where Cg is the center of gravity of the benzimidazole ring) for the centroid···centroid separations of rings N1 / N2 / C1— C7 and N1 / N2 / C1— C7(symmetry code: 1-x, y, 3/2-z). The three-dimensional framework is defined by intermolecular hydrogen bonds involving water molecules, the uncoordinated imidazole N atoms, and carboxylate O atoms(N—H···O and O—H···O, Table 2 and Fig. 2).

### Experimental

1*H*-benzo[d]imidazole-2-carboxylic acid(20 mg, 0.12 mmol) and zinc chloride dihydrate (10 mg, 0.06 mmol) were separately dissolved in 5 ml methanol. The solutions were mixed and stirred magnetically for 2 h. Colourless single crystals were isolated from the solution at room temperature over several days.

### Refinement

At first, all H atoms were located from the difference Fourier maps, and then were treated as riding [C—H = 0.93 Å ; U_iso_(H) = 1.2 times U_eq_; imidazole N—H = 0.86 Å and O—H = 0.85 Å; U_iso_(H) = 1.5 times U_eq_]. The weighting schemes were optimized .

### Crystal data

**Table:**

[Zn(C_8_H_5_N_2_O_2_)_2_(H_2_O)]	*F*(000) = 1648
*M**_r_* = 405.67	*D*_x_ = 1.581 Mg m^−^^3^
Monoclinic, *C*2/*c*	Mo *K*α radiation, λ = 0.71073 Å
Hall symbol: -C 2yc	Cell parameters from 25 reflections
*a* = 25.9702 (15) Å	θ = 10–13°
*b* = 10.0870 (6) Å	µ = 1.48 mm^−^^1^
*c* = 16.7885 (10) Å	*T* = 293 K
β = 129.21 (3)°	Block, colourless
*V* = 3407.8 (14) Å^3^	0.20 × 0.10 × 0.10 mm
*Z* = 8	

### Data collection

**Table:**

Enraf–Nonius CAD-4 diffractometer	2368 reflections with *I* > 2σ(*I*)
Radiation source: fine-focus sealed tube	*R*_int_ = 0.039
graphite	θ_max_ = 25.3°, θ_min_ = 2.0°
ω/2θ scans	*h* = −31→31
Absorption correction: ψ scan (*ABSCOR*; Higashi, 1995)	*k* = 0→12
*T*_min_ = 0.757, *T*_max_ = 0.866	*l* = −20→20
6178 measured reflections	3 standard reflections every 120 min
3097 independent reflections	intensity decay: 1%

### Refinement

**Table:**

Refinement on *F*^2^	Primary atom site location: structure-invariant direct methods
Least-squares matrix: full	Secondary atom site location: difference Fourier map
*R*[*F*^2^ > 2σ(*F*^2^)] = 0.039	Hydrogen site location: inferred from neighbouring sites
*wR*(*F*^2^) = 0.101	H-atom parameters constrained
*S* = 0.99	*w* = 1/[σ^2^(*F*_o_^2^) + (0.056*P*)^2^] where *P* = (*F*_o_^2^ + 2*F*_c_^2^)/3
3097 reflections	(Δ/σ)_max_ = 0.001
235 parameters	Δρ_max_ = 0.56 e Å^−^^3^
0 restraints	Δρ_min_ = −0.56 e Å^−^^3^

### Special details

**Table:**

Geometry. All esds (except the esd in the dihedral angle between two l.s. planes) are estimated using the full covariance matrix. The cell esds are taken into account individually in the estimation of esds in distances, angles and torsion angles; correlations between esds in cell parameters are only used when they are defined by crystal symmetry. An approximate (isotropic) treatment of cell esds is used for estimating esds involving l.s. planes.
Refinement. Refinement of *F*^2^ against ALL reflections. The weighted *R*-factor wR and goodness of fit *S* are based on *F*^2^, conventional *R*-factors *R* are based on *F*, with *F* set to zero for negative *F*^2^. The threshold expression of *F*^2^ > σ(*F*^2^) is used only for calculating *R*-factors(gt) etc. and is not relevant to the choice of reflections for refinement. *R*-factors based on *F*^2^ are statistically about twice as large as those based on *F*, and *R*- factors based on ALL data will be even larger.

### Fractional atomic coordinates and isotropic or equivalent isotropic displacement parameters (Å^2^)

**Table:**

	*x*	*y*	*z*	*U*_iso_*/*U*_eq_	
Zn1	0.569627 (17)	0.05468 (4)	0.69627 (3)	0.02900 (14)	
O1	0.50351 (16)	0.3274 (3)	0.4673 (2)	0.0650 (9)	
O2	0.54498 (12)	0.1385 (2)	0.55532 (17)	0.0374 (6)	
O3	0.67653 (12)	−0.1506 (2)	0.97193 (17)	0.0413 (6)	
O4	0.60004 (11)	−0.0175 (2)	0.84308 (17)	0.0364 (6)	
O5	0.48517 (12)	−0.0394 (3)	0.61712 (19)	0.0544 (8)	
H5A	0.4577	−0.0325	0.5520	0.065*	
H5B	0.4758	−0.0872	0.6481	0.065*	
N1	0.56695 (13)	0.2485 (3)	0.7205 (2)	0.0330 (6)	
N2	0.54861 (16)	0.4515 (3)	0.6583 (2)	0.0433 (8)	
H2A	0.5375	0.5146	0.6156	0.052*	
N3	0.64903 (12)	−0.0562 (3)	0.74399 (19)	0.0299 (6)	
N4	0.72922 (13)	−0.1867 (3)	0.8647 (2)	0.0392 (7)	
H4A	0.7536	−0.2320	0.9206	0.047*	
C1	0.6165 (2)	0.4261 (5)	0.9534 (3)	0.0632 (12)	
H1A	0.6323	0.4153	1.0204	0.076*	
C2	0.6052 (2)	0.5536 (5)	0.9134 (3)	0.0622 (12)	
H2B	0.6135	0.6254	0.9547	0.075*	
C3	0.5824 (2)	0.5773 (4)	0.8158 (3)	0.0567 (11)	
H3A	0.5750	0.6628	0.7900	0.068*	
C4	0.57069 (19)	0.4657 (3)	0.7569 (3)	0.0422 (9)	
C5	0.58192 (16)	0.3372 (4)	0.7962 (3)	0.0353 (8)	
C6	0.60498 (19)	0.3157 (4)	0.8957 (3)	0.0466 (9)	
H6A	0.6123	0.2305	0.9222	0.056*	
C7	0.54749 (17)	0.3217 (3)	0.6408 (3)	0.0351 (8)	
C8	0.52981 (18)	0.2603 (4)	0.5450 (3)	0.0377 (8)	
C9	0.64989 (16)	−0.0943 (3)	0.8895 (2)	0.0309 (7)	
C10	0.67732 (15)	−0.1125 (3)	0.8339 (2)	0.0295 (7)	
C11	0.68567 (15)	−0.0954 (4)	0.7139 (2)	0.0318 (8)	
C12	0.67916 (17)	−0.0663 (4)	0.6270 (3)	0.0415 (9)	
H12A	0.6452	−0.0120	0.5753	0.050*	
C13	0.72500 (19)	−0.1208 (4)	0.6205 (3)	0.0514 (11)	
H13A	0.7224	−0.1014	0.5640	0.062*	
C14	0.7749 (2)	−0.2041 (5)	0.6966 (3)	0.0587 (12)	
H14A	0.8042	−0.2410	0.6885	0.070*	
C15	0.78262 (19)	−0.2338 (4)	0.7829 (3)	0.0547 (11)	
H15A	0.8167	−0.2886	0.8341	0.066*	
C16	0.73686 (17)	−0.1779 (4)	0.7905 (3)	0.0378 (8)	

### Atomic displacement parameters (Å^2^)

**Table:**

	*U*^11^	*U*^22^	*U*^33^	*U*^12^	*U*^13^	*U*^23^
Zn1	0.0261 (2)	0.0311 (2)	0.0279 (2)	0.00695 (17)	0.01612 (17)	0.00661 (17)
O1	0.110 (3)	0.0434 (17)	0.0441 (16)	0.0153 (17)	0.0498 (18)	0.0146 (14)
O2	0.0499 (14)	0.0327 (14)	0.0329 (13)	0.0076 (11)	0.0277 (12)	0.0067 (11)
O3	0.0463 (14)	0.0491 (16)	0.0300 (13)	0.0162 (12)	0.0248 (12)	0.0143 (12)
O4	0.0365 (13)	0.0456 (15)	0.0319 (12)	0.0156 (11)	0.0238 (11)	0.0129 (11)
O5	0.0410 (14)	0.089 (2)	0.0384 (15)	−0.0239 (15)	0.0277 (13)	−0.0217 (14)
N1	0.0356 (15)	0.0309 (15)	0.0288 (15)	0.0036 (12)	0.0187 (13)	0.0037 (12)
N2	0.0580 (19)	0.0310 (17)	0.0414 (17)	0.0026 (15)	0.0316 (16)	0.0080 (14)
N3	0.0272 (14)	0.0362 (15)	0.0250 (14)	0.0105 (12)	0.0159 (12)	0.0095 (12)
N4	0.0362 (16)	0.0500 (19)	0.0305 (15)	0.0204 (14)	0.0206 (14)	0.0189 (14)
C1	0.068 (3)	0.075 (3)	0.044 (2)	−0.011 (3)	0.034 (2)	−0.014 (2)
C2	0.074 (3)	0.054 (3)	0.056 (3)	−0.014 (2)	0.041 (3)	−0.025 (2)
C3	0.070 (3)	0.038 (2)	0.063 (3)	−0.007 (2)	0.042 (3)	−0.011 (2)
C4	0.047 (2)	0.035 (2)	0.045 (2)	−0.0039 (16)	0.0293 (19)	−0.0041 (17)
C5	0.0328 (18)	0.039 (2)	0.0334 (19)	0.0000 (16)	0.0204 (16)	−0.0015 (16)
C6	0.052 (2)	0.048 (2)	0.034 (2)	0.0009 (19)	0.0247 (19)	0.0001 (18)
C7	0.041 (2)	0.0280 (19)	0.036 (2)	0.0033 (15)	0.0245 (17)	0.0047 (16)
C8	0.045 (2)	0.037 (2)	0.0331 (19)	0.0056 (17)	0.0257 (17)	0.0087 (16)
C9	0.0310 (18)	0.0343 (18)	0.0245 (17)	0.0022 (15)	0.0161 (15)	0.0026 (15)
C10	0.0247 (16)	0.0313 (17)	0.0285 (17)	0.0064 (14)	0.0149 (14)	0.0045 (15)
C11	0.0259 (16)	0.0411 (19)	0.0263 (17)	0.0038 (14)	0.0156 (15)	0.0011 (15)
C12	0.0363 (19)	0.054 (2)	0.0334 (19)	0.0130 (18)	0.0216 (17)	0.0142 (18)
C13	0.046 (2)	0.074 (3)	0.042 (2)	0.014 (2)	0.032 (2)	0.013 (2)
C14	0.046 (2)	0.086 (3)	0.059 (3)	0.026 (2)	0.040 (2)	0.017 (2)
C15	0.042 (2)	0.076 (3)	0.049 (2)	0.031 (2)	0.031 (2)	0.024 (2)
C16	0.0327 (18)	0.047 (2)	0.0341 (19)	0.0130 (16)	0.0212 (16)	0.0104 (17)

### Geometric parameters (Å, °)

**Table:**

Zn1—O5	1.947 (3)	C1—C2	1.393 (6)
Zn1—N1	2.007 (3)	C1—H1A	0.9300
Zn1—N3	2.014 (2)	C2—C3	1.365 (6)
Zn1—O4	2.184 (2)	C2—H2B	0.9300
Zn1—O2	2.193 (2)	C3—C4	1.400 (5)
O1—C8	1.224 (4)	C3—H3A	0.9300
O2—C8	1.268 (4)	C4—C5	1.398 (5)
O3—C9	1.227 (4)	C5—C6	1.390 (5)
O4—C9	1.268 (4)	C6—H6A	0.9300
O5—H5A	0.8500	C7—C8	1.500 (5)
O5—H5B	0.8500	C9—C10	1.502 (4)
N1—C7	1.318 (4)	C11—C12	1.389 (5)
N1—C5	1.394 (4)	C11—C16	1.397 (5)
N2—C7	1.338 (4)	C12—C13	1.377 (5)
N2—C4	1.375 (5)	C12—H12A	0.9300
N2—H2A	0.8600	C13—C14	1.387 (5)
N3—C10	1.318 (4)	C13—H13A	0.9300
N3—C11	1.387 (4)	C14—C15	1.366 (5)
N4—C10	1.328 (4)	C14—H14A	0.9300
N4—C16	1.383 (4)	C15—C16	1.391 (5)
N4—H4A	0.8600	C15—H15A	0.9300
C1—C6	1.379 (6)		
			
O5—Zn1—N1	116.39 (12)	C3—C4—C5	121.7 (4)
O5—Zn1—N3	115.42 (12)	N1—C5—C6	131.0 (3)
N1—Zn1—N3	128.19 (11)	N1—C5—C4	108.1 (3)
O5—Zn1—O4	93.18 (11)	C6—C5—C4	120.9 (3)
N1—Zn1—O4	97.49 (10)	C1—C6—C5	117.1 (4)
N3—Zn1—O4	79.46 (9)	C1—C6—H6A	121.5
O5—Zn1—O2	90.98 (11)	C5—C6—H6A	121.5
N1—Zn1—O2	79.56 (10)	N1—C7—N2	112.6 (3)
N3—Zn1—O2	99.82 (10)	N1—C7—C8	121.2 (3)
O4—Zn1—O2	175.67 (9)	N2—C7—C8	126.1 (3)
C8—O2—Zn1	112.0 (2)	O1—C8—O2	126.7 (3)
C9—O4—Zn1	113.4 (2)	O1—C8—C7	120.0 (3)
Zn1—O5—H5A	120.4	O2—C8—C7	113.3 (3)
Zn1—O5—H5B	119.6	O3—C9—O4	127.2 (3)
H5A—O5—H5B	120.0	O3—C9—C10	119.6 (3)
C7—N1—C5	105.8 (3)	O4—C9—C10	113.2 (3)
C7—N1—Zn1	112.4 (2)	N3—C10—N4	112.5 (3)
C5—N1—Zn1	141.7 (2)	N3—C10—C9	121.3 (3)
C7—N2—C4	107.5 (3)	N4—C10—C9	126.2 (3)
C7—N2—H2A	126.3	C12—C11—N3	131.7 (3)
C4—N2—H2A	126.3	C12—C11—C16	120.3 (3)
C10—N3—C11	106.3 (3)	N3—C11—C16	107.9 (3)
C10—N3—Zn1	112.6 (2)	C13—C12—C11	117.5 (3)
C11—N3—Zn1	141.2 (2)	C13—C12—H12A	121.3
C10—N4—C16	107.5 (3)	C11—C12—H12A	121.3
C10—N4—H4A	126.2	C12—C13—C14	121.4 (4)
C16—N4—H4A	126.2	C12—C13—H13A	119.3
C6—C1—C2	121.4 (4)	C14—C13—H13A	119.3
C6—C1—H1A	119.3	C15—C14—C13	122.3 (4)
C2—C1—H1A	119.3	C15—C14—H14A	118.9
C3—C2—C1	122.6 (4)	C13—C14—H14A	118.9
C3—C2—H2B	118.7	C14—C15—C16	116.6 (3)
C1—C2—H2B	118.7	C14—C15—H15A	121.7
C2—C3—C4	116.3 (4)	C16—C15—H15A	121.7
C2—C3—H3A	121.9	N4—C16—C15	132.3 (3)
C4—C3—H3A	121.9	N4—C16—C11	105.8 (3)
N2—C4—C3	132.3 (4)	C15—C16—C11	121.9 (3)
N2—C4—C5	106.0 (3)		
			
O5—Zn1—O2—C8	105.9 (2)	Zn1—N1—C7—N2	−177.1 (2)
N1—Zn1—O2—C8	−10.8 (2)	C5—N1—C7—C8	177.4 (3)
N3—Zn1—O2—C8	−138.1 (2)	Zn1—N1—C7—C8	0.2 (4)
O5—Zn1—O4—C9	113.9 (2)	C4—N2—C7—N1	0.3 (4)
N1—Zn1—O4—C9	−128.9 (2)	C4—N2—C7—C8	−176.9 (3)
N3—Zn1—O4—C9	−1.3 (2)	Zn1—O2—C8—O1	−166.9 (3)
O5—Zn1—N1—C7	−80.8 (3)	Zn1—O2—C8—C7	13.2 (4)
N3—Zn1—N1—C7	99.4 (2)	N1—C7—C8—O1	170.2 (3)
O4—Zn1—N1—C7	−178.1 (2)	N2—C7—C8—O1	−12.9 (6)
O2—Zn1—N1—C7	5.1 (2)	N1—C7—C8—O2	−9.9 (5)
O5—Zn1—N1—C5	103.6 (4)	N2—C7—C8—O2	167.0 (3)
N3—Zn1—N1—C5	−76.2 (4)	Zn1—O4—C9—O3	−178.6 (3)
O4—Zn1—N1—C5	6.3 (4)	Zn1—O4—C9—C10	2.2 (4)
O2—Zn1—N1—C5	−170.5 (4)	C11—N3—C10—N4	−0.7 (4)
O5—Zn1—N3—C10	−88.5 (2)	Zn1—N3—C10—N4	179.3 (2)
N1—Zn1—N3—C10	91.3 (3)	C11—N3—C10—C9	−178.7 (3)
O4—Zn1—N3—C10	0.0 (2)	Zn1—N3—C10—C9	1.3 (4)
O2—Zn1—N3—C10	175.6 (2)	C16—N4—C10—N3	0.9 (4)
O5—Zn1—N3—C11	91.5 (4)	C16—N4—C10—C9	178.9 (3)
N1—Zn1—N3—C11	−88.7 (4)	O3—C9—C10—N3	178.3 (3)
O4—Zn1—N3—C11	180.0 (4)	O4—C9—C10—N3	−2.5 (5)
O2—Zn1—N3—C11	−4.4 (4)	O3—C9—C10—N4	0.5 (5)
C6—C1—C2—C3	−0.2 (7)	O4—C9—C10—N4	179.8 (3)
C1—C2—C3—C4	0.1 (7)	C10—N3—C11—C12	−179.6 (4)
C7—N2—C4—C3	179.0 (4)	Zn1—N3—C11—C12	0.4 (7)
C7—N2—C4—C5	−0.5 (4)	C10—N3—C11—C16	0.1 (4)
C2—C3—C4—N2	−179.5 (4)	Zn1—N3—C11—C16	−179.8 (3)
C2—C3—C4—C5	−0.1 (6)	N3—C11—C12—C13	179.3 (4)
C7—N1—C5—C6	−179.6 (4)	C16—C11—C12—C13	−0.4 (5)
Zn1—N1—C5—C6	−3.8 (6)	C11—C12—C13—C14	1.4 (6)
C7—N1—C5—C4	−0.4 (4)	C12—C13—C14—C15	−1.8 (7)
Zn1—N1—C5—C4	175.3 (3)	C13—C14—C15—C16	1.1 (7)
N2—C4—C5—N1	0.6 (4)	C10—N4—C16—C15	179.6 (4)
C3—C4—C5—N1	−179.0 (3)	C10—N4—C16—C11	−0.8 (4)
N2—C4—C5—C6	179.9 (3)	C14—C15—C16—N4	179.4 (4)
C3—C4—C5—C6	0.3 (6)	C14—C15—C16—C11	−0.1 (6)
C2—C1—C6—C5	0.4 (6)	C12—C11—C16—N4	−179.9 (3)
N1—C5—C6—C1	178.6 (4)	N3—C11—C16—N4	0.4 (4)
C4—C5—C6—C1	−0.5 (6)	C12—C11—C16—C15	−0.2 (6)
C5—N1—C7—N2	0.1 (4)	N3—C11—C16—C15	−180.0 (4)

### Hydrogen-bond geometry (Å, °)

**Table:**

*D*—H···*A*	*D*—H	H···*A*	*D*···*A*	*D*—H···*A*
N2—H2A···O1^i^	0.86	1.93	2.765 (4)	162
N4—H4A···O3^ii^	0.86	1.95	2.778 (4)	161
O5—H5A···O2^iii^	0.85	2.06	2.670 (4)	128
O5—H5B···O4^iv^	0.85	2.18	2.695 (5)	119

Symmetry codes: (i) −*x*+1, −*y*+1, −*z*+1; (ii) −*x*+3/2, −*y*−1/2, −*z*+2; (iii) −*x*+1, −*y*, −*z*+1; (iv) −*x*+1, *y*, −*z*+3/2.
